# Factors associated with access channels and satisfaction with health science popularization among health lifestyle instructors

**DOI:** 10.3389/fpubh.2026.1874724

**Published:** 2026-07-29

**Authors:** Xu-peng Chen, Su-su Zhuang, Ting Zhang, Li-wen Wei, Shi-ya Ji, Li-li Gou, Hua-feng Yang

**Affiliations:** Nanjing Municipal Center for Disease Control and Prevention, Nanjing, China

**Keywords:** access channels, health lifestyle instructors, health science popularization, influencing factors, mediating effect

## Abstract

**Background:**

Health Lifestyle Instructors (HLIs) play a pivotal role in community health promotion. The quality of and satisfaction with their own access to health science information may directly influence their work effectiveness, which in turn affect residents’ health literacy and the outcomes of chronic disease prevention and control. This cross-sectional questionnaire-based study aimed to (1) explore factors associated with satisfaction with access channels for health science popularization among HLIs and (2) examine whether self-rated health status statistically mediated the association between Healthy Cell Project (HCP) establishment and satisfaction with access channels. The findings were intended to provide an evidence base for optimizing HLIs training programs.

**Methods:**

Access channels for health science popularization were identified through multiple-response analysis. Potential factors associated with satisfaction with these access channels were first screened using univariate analysis, followed by logistic regression analysis. The mediating role of self-rated health status was examined using the SPSS PROCESS macro with the bootstrap method.

**Results:**

A total of 3,032 valid questionnaires were analyzed. Online searches and community publicity constituted the primary access channels for health science information. Among new media platforms, WeChat official accounts and short-video applications were the most frequently used. Significantly higher satisfaction with access channels was observed among HLIs who reported better self-rated health, worked in township/community/village units, or were affiliated with institutions that had established the HCP (all *p* < 0.01). Significant correlations emerged among self-rated health, the establishment of the HCP, and satisfaction with access channels (*r* = 0.085–0.135, *p* < 0.01). Mediation analysis suggested that self-rated health status partially mediated the association between HCP establishment and satisfaction with access channels (effect size = 0.063, 95% CI: 0.031–0.104), although the explained variance was small (*R^2^ =* 0.017).

**Conclusion:**

Access channels for health science popularization among HLIs were characterized primarily by online - offline integration, with differences in satisfaction across groups. Self-rated health status partially mediated the association between HCP establishment and satisfaction with access channels in this cross-sectional analysis.

## Introduction

Health education serves as a cornerstone of public health practice and is pivotal in both chronic disease prevention and control and the improvement of population health outcomes (1, 2). As a critical link between the professional health system and community residents, public health workers translate complex health knowledge into actionable behaviors for the public, making them a central force in grassroots health promotion (3). In China, Health Lifestyle Instructors (HLIs) are a unique group of Community Health Workers (CHWs) developed under national public health strategies. They are volunteers or social workers with basic health knowledge and skills who provide health education and guidance at the family and community levels. Closely connected to local communities, HLIs serve as the main implementers of the China Healthy Lifestyle for All campaign, a national public health initiative launched in 2007. The campaign emphasizes key areas such as balanced nutrition and regular physical activity, with the goal of enhancing national health literacy and reducing the burden of chronic non-communicable diseases. The quality of the health science information they access, together with their ability to apply it, directly affects the professionalism of the community health guidance services they provide (4–6). With the advancement of the China Healthy Lifestyle for All campaign, research on HLIs has also expanded. Recent studies have primarily focused on HLIs’ willingness to perform their duties, the effectiveness of health guidance evaluations, and the development of intervention models (7–11). However, systematic research on how HLIs access health science information remains limited, although existing studies suggest that satisfaction with such access is shaped by multiple factors. Key determinants include an individual health status, healthcare background, educational attainment, and income (12, 13). Nevertheless, the primary channels through which HLIs access health science information remain unclear. Furthermore, a systematic analysis is lacking regarding the key factors that influence their satisfaction with the information acquired through these channels.

CHWs are widely recognized as trusted community members who provide health education and basic care and perform roles comparable to those of HLIs in China (14). International research on CHWs began earlier and is broader in scope. Existing studies have shown that CHWs play an important role in improving population health, strengthening supportive health environments, reducing disparities, and serving vulnerable communities (15–18). In economically developed countries and regions, CHWs primarily access health information through professional training, collaboration with medical institutions, and government-funded platforms (19, 20). In low-income and middle-income countries (LMICs), however, CHWs are frequently hindered by limited training opportunities and insufficient supportive supervision (21–23). Regarding mediating mechanisms, previous studies have identified that self-rated health as an individual cognitive factor that may mediate the association between supportive environments and health information-seeking behavior, thereby providing theoretical basis for further exploration of related mechanisms (24–27). Compared with international research, relevant studies in China show both similarities and differences. A major similarity is that both HLIs in China and CHWs in other settings are largely non-physician community-based health workers whose effectiveness depends on the quality of the health information they receive and the training they undergo. Moreover, the effectiveness of information access is shaped by both the supportive environment and individual characteristics (28). Differences lie in training models and research priorities. HLIs in China are driven by national policy, with standardized training protocols and large-scale implementation, whereas CHWs training in many other countries is community-led and supported by diverse funding sources (23). China places greater emphasis on the effectiveness of health interventions, while international studies often focus more on equity issues and technical support (23, 29). Furthermore, international research has advanced further in examining the mediating mechanisms underlying health information access. In contrast, domestic studies investigating the mediating role of individual cognitive factors in the relationship between supportive environments and satisfaction with health science information access remain at an early stage (28).

The Healthy Cell Project (HCP) is a health-supportive initiative designed to disseminate health knowledge, promote health skills, and foster a culture of healthy lifestyles at the community level (30). It includes a range of health-promoting settings, such as healthy communities, workplaces, schools, canteens, restaurants, trails, huts, and theme parks. As such, it is an integral component of the national China Healthy Lifestyle for All campaign (31). Existing studies suggest that satisfaction with health science information is influenced not by a single factor but by the combined effects of multiple dimensions, including individual characteristics, environmental support, and cognitive levels (12). Social cognitive theory posits that external environmental factors may shape an individual’ s psychological and cognitive processes, which are in turn related to behavioral outcomes and perceptions of needs (26, 27, 32). Within this theoretical framework, the HCP may represent an important health-supportive environment at the community level. Its establishment may be associated with better access to health science information among HLIs by fostering a supportive atmosphere for health communication and improving the accessibility of science popularization resources. As a key subjective indicator of personal health perception, self-rated health may serve as a mediator between environmental support and the satisfaction of health-related needs. Specifically, the supportive environment related to HCP establishment may be associated with better health cognition among HLIs, which in turn may be linked to greater satisfaction with access to health science information (33, 34).

This study aimed to provide empirical evidence for optimizing HLIs training systems, improving the allocation of health science popularization resources, and strengthening the professional capacity of grassroots HLIs. Furthermore, it sought to provide practical support for advancing the China Healthy Lifestyle for All campaign and contributing to the construction of a Healthy China.

## Objects and methods

### Study design and participants

This study was a cross-sectional, questionnaire-based survey. All HLIs recruited in Nanjing in September 2022 and September 2023 who had completed the training course and passed the assessment were included in the study. The survey was conducted in September 2022 and September 2023, coinciding with the National Healthy Lifestyle Month in China.

### Survey method

HLIs were recruited within the routine training framework of the Nanjing Health Lifestyle Instructor program through voluntary registration and local recommendation by community or institutional organizations. This was a non-probability sampling process rather than a probability-based sampling design. Eligible participants included community backbone members and grassroots cadres, social sports instructors, primary healthcare personnel, residents committed to public welfare, and staff members from various organizations. Participants were required to have healthy lifestyle behaviors as well as basic communication and coordination abilities. Each district recruited approximately 100–150 HLIs. All enrolled HLIs completed the Nanjing Health Lifestyle Instructor training course and passed the assessment before receiving certification. The training was delivered by provincial - and municipal - level experts in public health communication, nutrition, physical activity, traditional Chinese medicine health preservation, and chronic disease prevention and control. After completion of the training, participants were invited to complete the online questionnaire.

This study was approved by the Ethics Committee of the Nanjing Municipal Center for Disease Control and Prevention (Ethical Approval No. PJ2023-A001-01), and all respondents provided written informed consent before participation.

### Questionnaire content

The questionnaire used in this study was self-developed for routine public health monitoring purposes (see Supplementary File S1 for details). It comprised two sections: (1) basic information, including gender, age, educational level, height, weight, and other demographic characteristics; and (2) access channels for health science popularization, adapted from the health information-seeking behavior section of the Jiangsu Provincial Health Literacy Monitoring Questionnaire, an officially developed instrument used in routine public health surveillance in Jiangsu Province, China.

The questionnaire was not a validated psychometric scale. It was developed on the basis of routine surveillance practice and was not subjected to a separate pilot test or formal content validity evaluation before use in the present study. HCP establishment was assessed by a self-reported item asking whether the respondent’ s affiliated work unit had established the HCP, with three response options: “Yes”, “No”, and “Don’ t know”. Self-rated health status was measured using five ordered response categories: “Very poor”, “Poor”, “General”, “Good”, and “Very good”. Satisfaction with access channels for health science popularization was assessed using a single-item dichotomous question with two response options: “satisfied” and “dissatisfied”.

### Quality control

All respondents completed the questionnaire independently, and each IP address was restricted to a single submission to prevent duplicate responses. Missing item checks were implemented on the electronic platform to minimize missing data. Questionnaire completion time was monitored as part of quality control, and questionnaires completed in less than 60 s were considered unrealistically short and excluded. In addition, the research team reviewed returned questionnaires for completeness and logical consistency. Questionnaires with missing, inconsistent, duplicate, or otherwise invalid responses were excluded and, when feasible, returned to the respondents for completion or correction.

### Measurement of health science popularization access channels

In the multiple-response analysis, the selection proportion was defined as the percentage of total selections accounted for by each channel. Thus, the selection proportion reflected the relative share of each option among all selected options and summed to 100% across channels. This measure is appropriate for comparing the relative popularity of different channels. By contrast, prevalence rate refers to the proportion of participants who selected a given option among all survey respondents and is more suitable for evaluating the overall level of use of a channel in the study population. The same method for calculating selection proportion was applied to the items asking respondents to identify the most authoritative and most convenient channels. The authority selection proportion and convenience selection proportion were used as quantitative indicators for comparing channels and constructing the convenience-authority quadrant analysis.

### Statistical analysis

IBM SPSS (version 25.0) was used for all statistical analysis. Categorical variables were described as frequencies and percentages [*n* (%)]. Group differences in satisfaction with access channels for health science popularization were examined using the chi-square test, and trend chi-square tests were used for ordinal variables where appropriate. A two - sided *p* < 0.05 was considered statistically significant.

Variables showing statistically significant differences in univariate analysis were entered into a multivariable logistic regression model. Satisfaction with access channels for health science popularization was treated as the dependent variable and coded as 1 = satisfied and 0 = dissatisfied. In the logistic regression model, age, self-rated health status, work unit, medical and health background, and HCP establishment were entered as categorical variables. HCP establishment included three response categories (“yes” “no” and “don’ t know”), with “no” used as the reference category. Reference categories for the remaining independent variables are shown in Table 1. Odds ratios (ORs) and 95% confidence intervals (CIs) were reported.

**Table 1:** Analysis of influencing factors of satisfaction with health science popularization access channels.

Characteristic	β	S.E.	Wald *χ*^2^	*p*	OR (95% CI)
Age (years)					
18–29^a^					
30–39	−0.067	0.145	0.212	0.645	0.936 (0.705, 1.242)
40–49	−0.172	0.173	0.982	0.322	0.842 (0.600, 1.183)
≥50	−0.319	0.243	1.715	0.190	0.727 (0.451, 1.171)
Self-rated health status					
Very poor^a^					
Poor	0.809	0.609	1.764	0.184	2.245 (0.681, 7.407)
General	1.357	0.472	8.256	0.004	3.884 (1.539, 9.798)
Good	1.820	0.475	14.704	<0.001	6.169 (2.434, 15.636)
Very good	2.405	0.492	23.845	<0.001	11.077 (4.219, 29.080)
Work unit					
Town/Community/Village^a^					
Municipal/District patriotic health campaign office/CDC/Health promotion center	−0.479	0.282	2.872	0.090	0.620 (0.356, 1.078)
Secondary/Tertiary hospital	−0.529	0.297	3.182	0.074	0.589 (0.329, 1.054)
Community health service center	−0.583	0.182	10.215	0.001	0.558 (0.390, 0.798)
School/Enterprise	−0.478	0.269	3.162	0.075	0.620 (0.366, 1.050)
Medical and health background					
None^a^					
Self-engaged	0.158	0.226	0.485	0.486	1.171 (0.751, 1.824)
Family member engaged	0.047	0.188	0.062	0.803	1.048 (0.726, 1.513)
Friend engaged	0.249	0.149	2.806	0.094	1.283 (0.959, 1.716)
HCP establishment					
No^a^					
Don’t know	0.522	0.396	1.737	0.188	1.685 (0.776, 3.661)
Yes	0.675	0.187	13.039	<0.001	1.964 (1.362, 2.834)

a Indicates the reference group.

Spearman’ s rank correlation analysis was used to examine the associations among HCP establishment, self-rated health status, and satisfaction with access channels for health science popularization.

Mediation analysis was performed using Model 4 of the PROCESS macro (version 3.5) for SPSS. For this analysis, only participants with definite responses regarding HCP establishment (“yes” or “no”) were included, and those who responded “don’ t know” were excluded. HCP establishment was treated as the independent variable, self-rated health status was entered as an ordered numeric variable coded from 1 (“very poor”) to 5 (“very good”), and satisfaction with access channels for health science popularization was treated as the dichotomous outcome variable. Based on prior studies and regression results, age, work unit, and medical and health background were included as covariates. The indirect effect was tested using 5,000 bootstrap samples and a 95% CI. Because the outcome variable was dichotomous and the study was cross-sectional, this analysis was used to explore a statistical mediation pattern within a regression-based framework, and the results were interpreted cautiously rather than causally. A mediation effect was considered statistically significant if the 95% CI did not include zero.

## Results

### Basic characteristics

Among the HLIs included in the study in Nanjing in 2022 and 2023, most were female (69.4%), aged 30–39 years (44.5%), had college or bachelor’ s degrees (92.3%), and were affiliated with township/community/village units (72.8%). Most participants were non-smokers (88.5%), had a normal body mass index (BMI) (60.8%), and self-rated their health status as “good” (40.0%). Additionally, 90.7% of the participants reported that their workplaces had established the HCP, and 73.4% either worked in the medical and health sector themselves or had family members or friends working in this field (Table 2).

**Table 2:** Characteristics of HLIs in Nanjing, 2022 and 2023.

Characteristic	2022 *n* (%)	2023 *n* (%)	Total *n* (%)
Gender			
Male	560 (34.1)	367 (26.4)	927 (30.6)
Female	1,080 (65.9)	1,025 (73.6)	2,105 (69.4)
Age (years)			
18–29	539 (32.9)	378 (27.2)	917 (30.2)
30–39	720 (43.9)	629 (45.2)	1,349 (44.5)
40–49	294 (17.9)	285 (20.5)	579 (19.1)
≥50	87 (5.3)	100 (7.2)	187 (6.2)
Educational level			
Junior high school or below	23 (1.4)	8 (0.6)	31 (1.0)
Senior high school/ Secondary technical school	77 (4.7)	63 (4.5)	140 (4.6)
College/bachelor’s degree	1,507 (91.9)	1,291 (92.7)	2,798 (92.3)
Master’s degree or above	33 (2.0)	30 (2.2)	63 (2.1)
Work unit			
Municipal/District patriotic health campaign office/CDC/Health promotion center	91 (5.5)	29 (2.1)	120 (4.0)
Secondary/Tertiary hospital	41 (2.5)	103 (7.4)	144 (4.7)
Community health service center	305 (18.6)	139 (10.0)	444 (14.6)
School/Enterprise	93 (5.7)	23 (1.7)	116 (3.8)
Town/Community/Village	1,110 (67.7)	1,095 (78.9)	2,208 (72.8)
Smoking status			
Smoker	236 (14.4)	114 (8.2)	350 (11.5)
Non-smoker	1,404 (85.6)	1,278 (91.8)	2,682 (88.5)
BMI category			
Underweight	130 (7.9)	99 (7.1)	229 (7.6)
Normal weight	969 (59.1)	873 (62.7)	1842 (60.8)
Overweight	423 (25.8)	339 (24.4)	762 (25.1)
Obese	118 (7.2)	81 (5.8)	199 (6.6)
Self-rated health status			
Very poor	12 (0.7)	8 (0.6)	20 (0.7)
Poor	19 (1.2)	16 (1.1)	35 (1.2)
General	552 (33.7)	488 (35.1)	1,040 (34.3)
Good	653 (39.8)	559 (40.2)	1,212 (40.0)
Very good	404 (24.6)	321 (23.1)	725 (23.9)
HCP establishment			
Yes	1,475 (89.9)	1,276 (91.7)	2,751 (90.7)
No	165 (10.1)	49 (3.5)	214 (7.1)
Don’ t know	——	67 (4.8)	67 (2.2)
Medical and health background			
Self-engaged	189 (11.5)	233 (16.7)	422 (13.9)
Family member engaged	265 (16.2)	198 (14.2)	463 (15.3)
Friend engaged	735 (44.8)	604 (43.4)	1,339 (44.2)
None	451 (27.5)	357 (25.6)	808 (26.6)

### Multiple-response analysis of access channels for health science popularization

**Figure 1:**
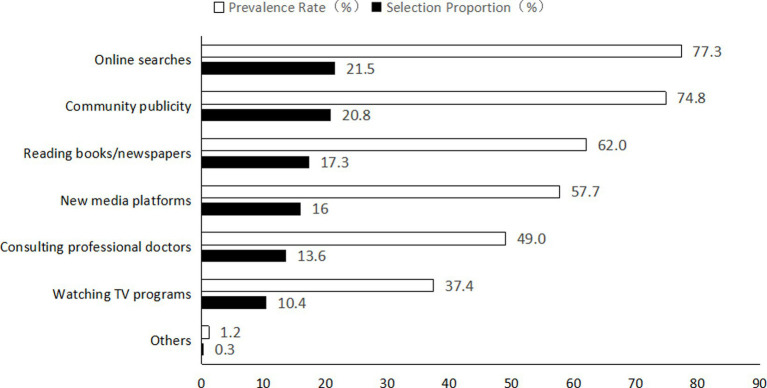
Health science popularization access channels among HLIs.

**Figure 2:**
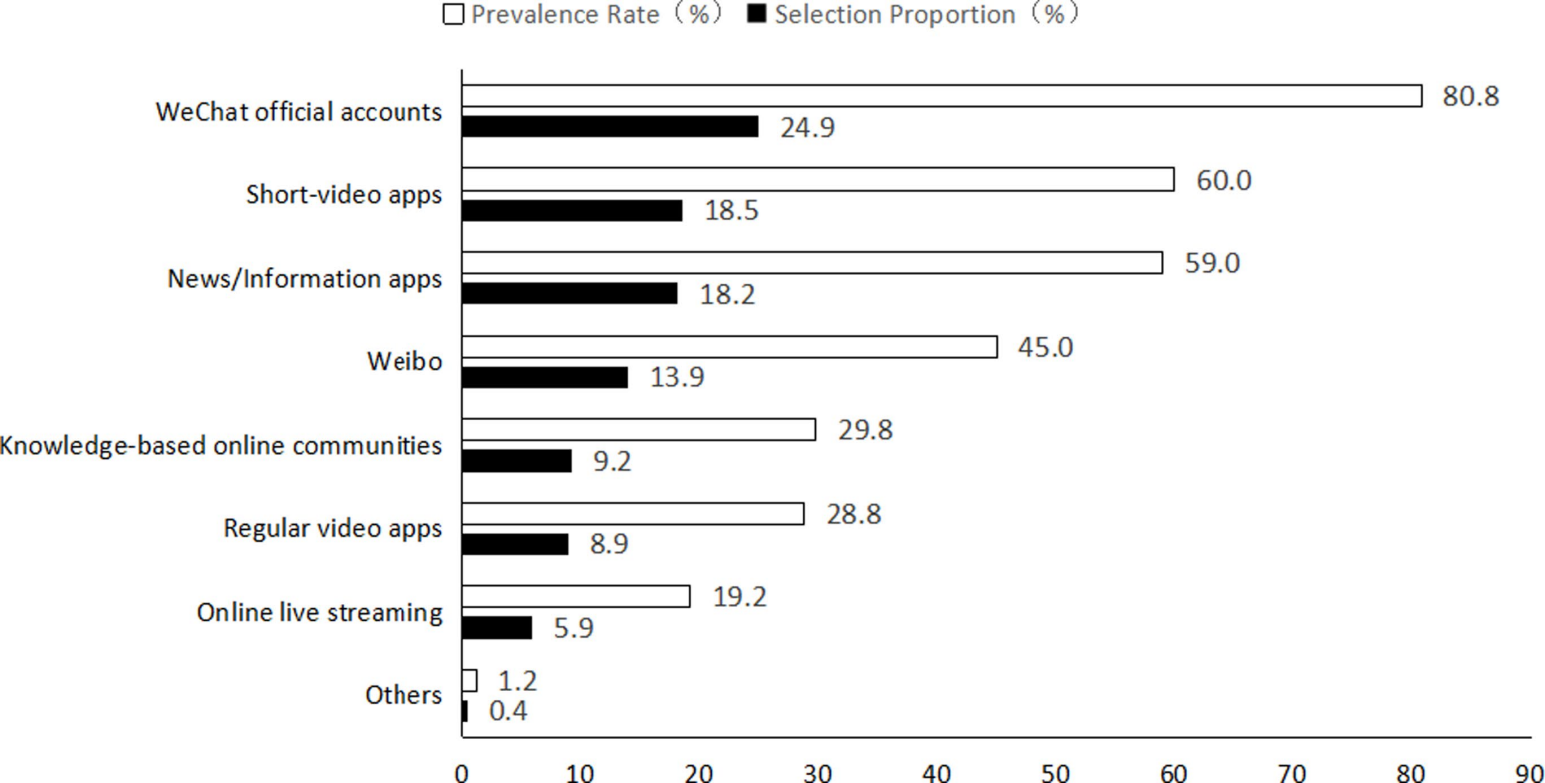
Health science popularization access channels on new media platforms.

Among HLIs, the three most commonly reported access channels for health science popularization were online searches (21.5%), community publicity (20.8%), and reading books/newspapers (17.3%). Among new media platforms, WeChat official accounts (24.9%), short-video apps (18.5%), and news/information applications (18.2%) were the most frequently utilized (Figures 1, 2).

**Figure 3:**
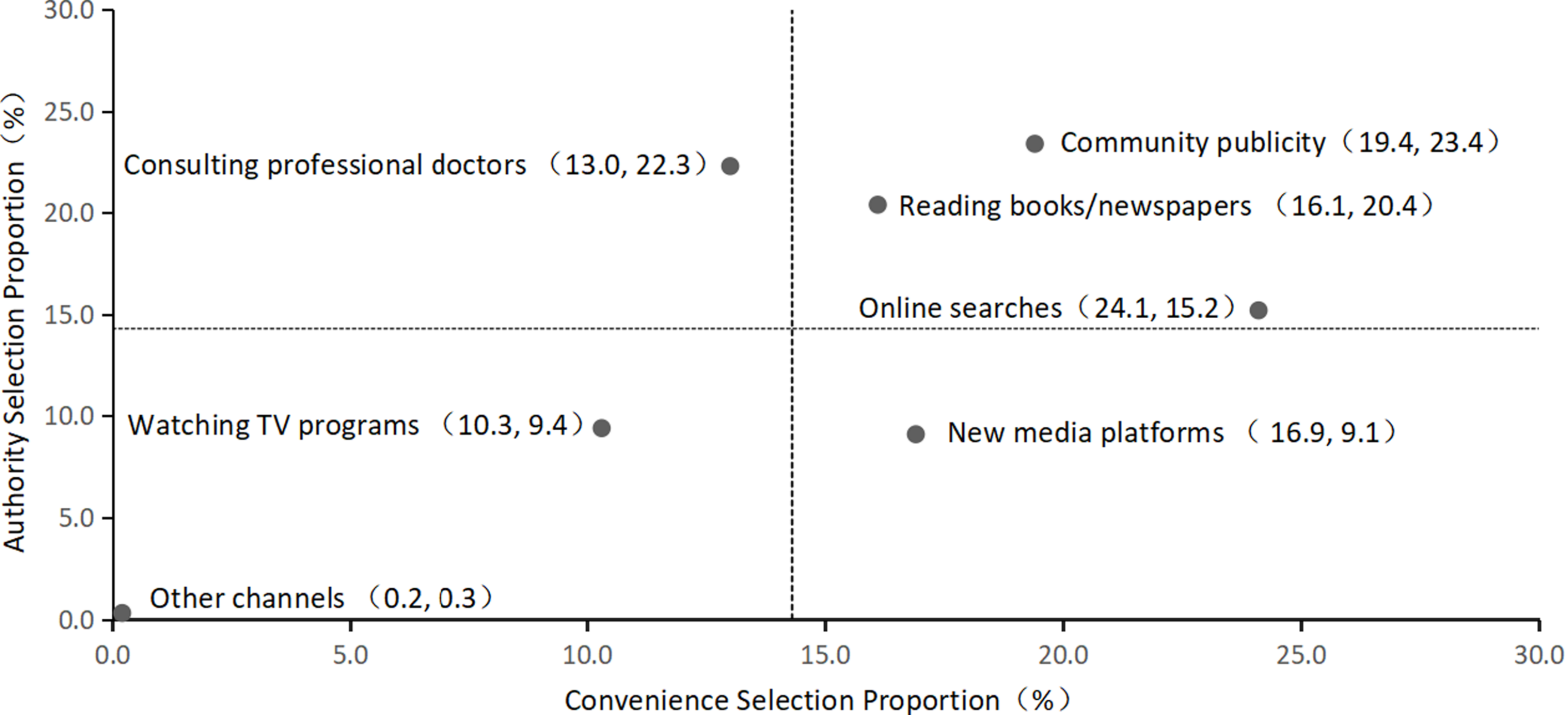
Distribution of health science popularization access channels by convenience and authority selection proportions.

**Figure 4:**
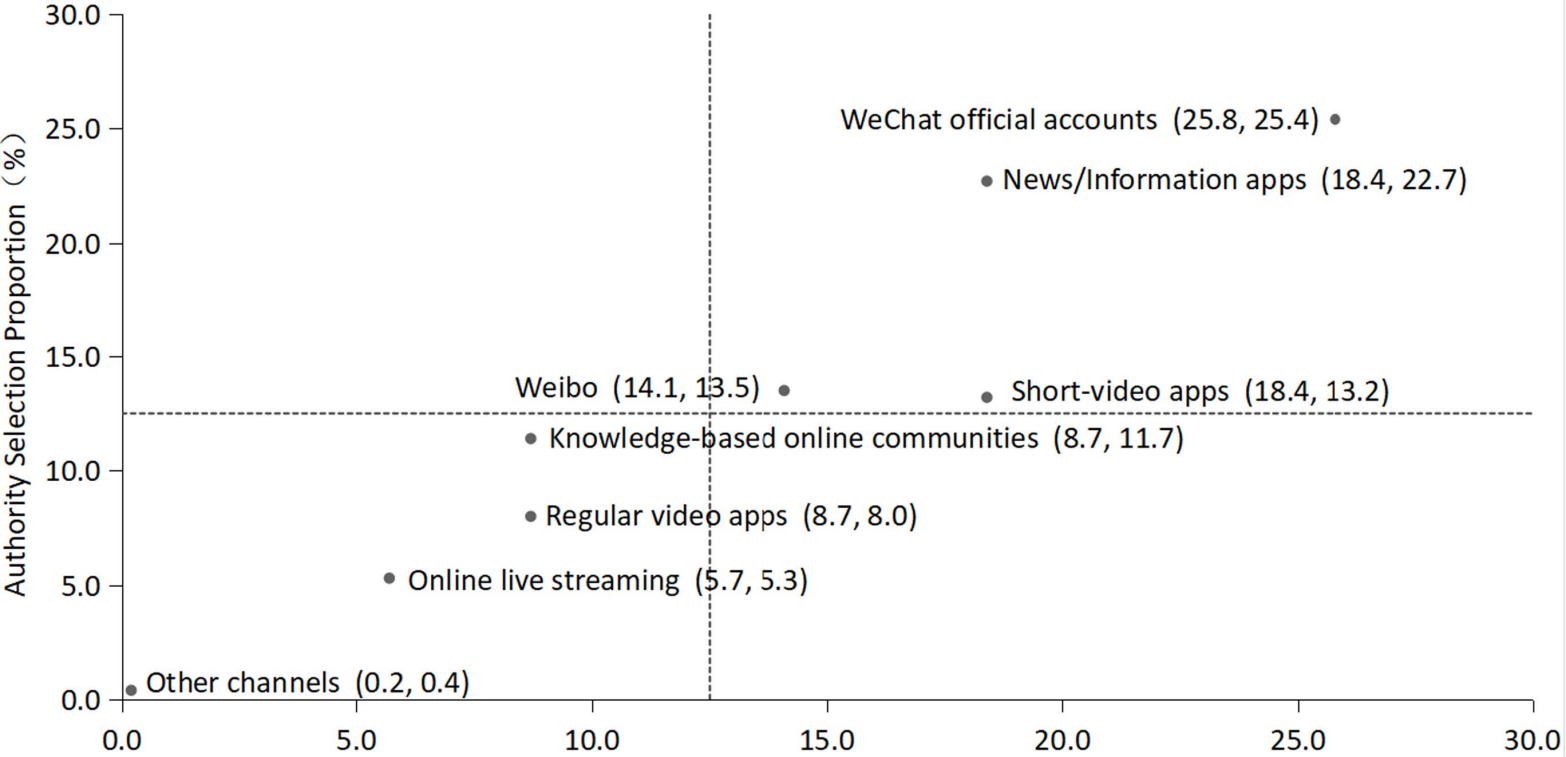
Distribution of new media health science popularization access channels by convenience and authority selection proportions.

To further examine the characteristics of these access channels, a scatter plot was constructed with the convenience selection proportion of each channel on the x-axis and the authority selection proportion on the y-axis. The mean values of the two proportions were used as cutoff points to divide channels into four quadrants: low convenience - low authority, high convenience - low authority, low convenience - high authority, and high convenience - high authority. The results showed that online searches had the highest convenience score among all channels (selection proportion: 24.1%), while community publicity had the highest authority score (selection proportion: 23.4%). Overall, reading books/newspapers, community publicity, and online searches showed a “high authority + high convenience profile”. Among new media platforms, WeChat official accounts, news/information applications, short-video applications, and Weibo also showed this dual-high profile, with WeChat official accounts appearing to provide the best balance between convenience and authority (Figures 3, 4).

### Satisfaction status of health science popularization access channels among HLIs with different characteristics

Satisfaction with access channels for health science popularization differed significantly according to work unit, medical and health background, and HCP establishment status (all *p* < 0.01). Satisfaction decreased with increasing age, and the trend was statistically significant (Trend *χ^2^* = 4.114, *p* < 0.05). By contrast, satisfaction increased with better self-rated health status, and this trend was also statistically significant (Trend *χ^2^* = 61.400, *p* < 0.01) (Table 3).

**Table 3:** Satisfaction status of health science popularization access channels among HLIs by different characteristics.

Characteristics	Category	Number of cases (Satisfaction rate, %)	*χ^2^* / Trend *χ^2^*	*p*
Gender	Male	836 (90.2)	1.858	0.173
	Female	1863 (88.5)		
Age (years)	18–29	826 (90.1)	4.114^a^	0.043
	30–39	1,205 (89.3)		
	40–49	508 (87.7)		
	≥50	160 (85.6)		
Education level	Junior high school or below	28 (90.3)	0.884^a^	0.347
	Senior high school/ Secondary technical school	126 (90.0)		
	College/bachelor’s degree	2,492 (89.1)		
	Master’s degree or above	53 (84.1)		
Work unit	Municipal/District patriotic health campaign office/CDC/Health promotion center	102 (85.0)	22.849	<0.001
	Secondary/Tertiary hospital	122 (84.7)		
	Community health service center	376 (84.7)		
	School/Enterprise	97 (83.6)		
	Town/Community/Village	2002 (90.7)		
Smoking status	Smoker	313 (89.4)	0.069	0.794
	Non-smoker	2,386 (89.0)		
BMI category	Underweight	204 (89.1)	0.110^a^	0.740
	Normal weight	1,642 (89.1)		
	Overweight	677 (88.8)		
	Obese	176 (88.4)		
Self-rated health status	Very poor	12 (60.0)	61.400^a^	<0.001
	Poor	26 (74.3)		
	General	883 (84.9)		
	Good	1,093 (90.2)		
	Very good	685 (94.5)		
Medical and health background	Self-engaged	357 (84.6)	15.533	0.001
	Family member engaged	412 (89.0)		
	Friend engaged	1,220 (91.1)		
	None	710 (87.9)		
HCP establishment	Yes	2,472 (89.9)	23.136	<0.001
	No	170 (79.4)		
	Don’ t know	57 (85.1)		

a Indicates the result of trend *χ*^2^ test.

### Analysis of influencing factors of satisfaction with health science popularization access channels

A multivariable logistic regression model was constructed using satisfaction with access channels for health science popularization as the dependent variable (1 = satisfied, 0 = dissatisfied). Variables that were statistically significant in univariate analysis - age, work unit, self-rated health status, medical and health background, and HCP establishment - were included as independent variables. Using “very poor” self-rated health status as the reference group, HLIs with “general”, “good”, and “very good” self-rated health status had significantly higher odds of being satisfied with access channels, with odds ratios (OR) of 3.884 (95% CI: 1.539–9.798), 6.169 (95% CI: 2.434–15.636), and 11.077 (95% CI: 4.219–29.080), respectively. Compared with HLIs affiliated with townships/communities/villages units, those working in community health service centers had lower odds of satisfaction with access channels (OR = 0.558, 95% CI: 0.390–0.798). In addition, HLIs working in units without HCP implementation had a lower satisfaction rate than those in units with HCP implementation (OR = 0.509, 95% CI: 0.353–0.734) (Table 1).

### Mediation effect analysis of self-rated health status

Spearman’ s rank correlation analysis showed positive correlations among the following three pairs of variables: HCP establishment and self-rated health status, HCP establishment and satisfaction with access channels for health science popularization, and self-rated health status and satisfaction with access channels for health science popularization. These findings suggest that HCP establishment was positively associated with both self-rated health status and satisfaction with access channels. In addition, HLIs with better self-rated health reported higher satisfaction, supporting the subsequent mediation analysis (Table 4).

**Table 4:** Correlations among HCP establishment, self-rated health status, and satisfaction with health science popularization access channels.

Variable	1	2	3
1. HCP establishment	1.000		
2. Self-rated health status	0.086^**^	1.000	
3. Satisfaction with health science popularization access channels	0.085^**^	0.135^**^	1.000

**indicates *p* < 0.01.

**Figure 5:**
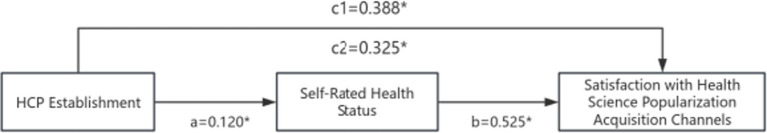
Statistical mediation of self-rated health status in the association between HCP establishment and satisfaction with access channels.

Based on the results of the preceding univariate and multivariate analyses, a mediation analysis was further performed to examine the mediating role of self-rated health status in the relationship between HCP establishment and satisfaction with access channels for health science popularization, with age, work unit, and medical and health background included as covariates. The mediation model was statistically significant (*F* = 12.838, *p* < 0.01), although the explained variance was small (*R^2^* = 0.017), indicating limited explanatory value. Specifically, HCP establishment was significantly associated with self-rated health status (*β* = 0.120, *t* = 4.286, *p* < 0.01). In turn, self-rated health status was significantly associated with satisfaction with access channels for health science popularization (*β* = 0.525, *Z* = 7.112, *p* < 0.01). The direct effect of HCP establishment on satisfaction with access channels was 0.325, accounting for 83.76% of the total effect. Self-rated health status showed a partial statistical mediation effect in this association, with an indirect effect of 0.063, accounted for 16.24% of the total effect. However, this finding should be interpreted cautiously because the cross-sectional design precludes causal inference and the model explained only a small proportion of the variance. Detailed results of the mediation analysis are presented in Table 5 and Figure 5.

**Table 5:** Statistical mediation of self-rated health status in the association between HCP establishment and satisfaction with health science popularization access channels.

Effect type	Path	Standard error	Effect value (95% CI)
Total effect	c1	0.093	0.388 (0.205, 0.571)
Direct effect	c2	0.091	0.325 (0.146, 0.504)
	a	0.028	0.120 (0.065, 0.175)
	b	0.074	0.525 (0.381, 0.670)
Mediating effect		0.018	0.063 (0.031, 0.104)

## Discussion

This study found that HLIs in Nanjing generally had relatively high educational attainment, with most holding college or bachelor’ s degrees. This profile is broadly consistent with international evidence on community-based health workers, suggesting that higher educational attainment may support stronger capacity for health communication and guidance (35, 36). We also found significant differences in satisfaction with access channels for health science popularization across work settings. Compared with HLIs affiliated with township/community/village units, those working in community health service centers reported lower satisfaction. One possible explanation is that grassroots health personnel often shoulder heavier routine service responsibilities while having relatively less access to authoritative, timely, and well-organized health information resources. Moreover, limitations in digital infrastructure, continuing education opportunities, and organizational support may further reduce the convenience and perceived usefulness of existing access channels (37).

This finding has important practical implications for HLIs training systems. Rather than adopting a one-size-fits-all approach, future HLIs programs should develop more targeted strategies for grassroots health personnel. For example, training content should be closely aligned with the practical demands of frontline work, and authoritative health science materials should be delivered through accessible and user-friendly channels. At the same time, greater efforts are needed to strengthen institutional support, improve the integration of online and offline learning resources, and enhance the efficiency of health information dissemination in grassroots settings. Such measures may help improve both satisfaction with access channels and the overall effectiveness of HLIs training.

This study found that although online searches were perceived as the most convenient channel for accessing health science information, traditional channels - including community publicity, consulting with healthcare professionals, and reading books or newspapers - remain the most authoritative sources (38). This tension between authority and convenience is common in the digital age (39, 40). International studies have similarly shown that although online health information is more convenient to access, people tend to place greater trust in information from official health institutions and healthcare professionals (41–43). Against this backdrop, future efforts should focus on narrowing the gap between authority and convenience. Specifically, this may be achieved by leveraging authoritative new media platforms, such as official WeChat accounts, and by involving healthcare professionals, researchers, and new media operators in the development and review of health science content (39). Such an approach may help promote health science communication that is both scientifically rigorous and publicly engaging.

A key finding of this study was that self-rated health status partially mediated the association between HCP establishment and satisfaction with access channels for health science popularization in the statistical model. This finding may have theoretical implications. First, the direct association between HCP establishment and satisfaction with access channels suggests that supportive environments may be associated with more favorable perceptions of access to health science popularization access (44). Second, the partial statistical mediation by self-rated health status indicates that individual health perception may also play a role in this association. HLIs with better self-rated health may also have greater self-efficacy, which may be related to more proactive and effective use of health information and, consequently, to higher satisfaction with access channels (45). This interpretation is consistent with international research identifying self-efficacy as an important factor influencing health information-seeking behavior (46).

However, given the cross-sectional design, these findings should be interpreted cautiously and should not be regarded as evidence of a confirmed causal pathway. In addition, the mediation model explained only a small proportion of the variance in satisfaction (*R^2^* = 0.017), suggesting limited practical significance and the likely involvement of other unmeasured individual, organizational, and contextual factors.

## Limitations

This study has several limitations. First, its cross-sectional design precludes determination of temporal sequence and limits causal inference regarding the associations among HCP establishment, self-rated health status, and satisfaction with access channels for health science popularization. Second, all variables were based on self-reported data and may therefore have been subject to reporting bias and social desirability bias. Third, the study was conducted in a single city (Nanjing), and participants were recruited through voluntary registration and local recommendation after completing training and certification. This recruitment process may have introduced selection bias and may limit the generalizability of the findings. Fourth, satisfaction was assessed using a single-item dichotomous measure, which may limit the depth and reliability of the outcome assessment and may not fully capture the multidimensional nature of satisfaction, such as the perceived quality, accuracy, convenience, authority, and usefulness of access channels for health science popularization. Finally, the mediation model explained only a small proportion of the variance in satisfaction (*R^2^* = 0.017), indicating limited explanatory power and practical significance. This suggests that satisfaction with access channels for health science popularization is likely influenced by additional individual, organizational, social, and contextual factors that were not captured in the present study.

## Conclusion

This study systematically examined factors associated with satisfaction with access channels for health science popularization among HLIs. The findings showed that the HLIs cohort in Nanjing was characterized by relatively high educational attainment, a strong grassroots orientation, generally healthy behaviors, substantial exposure to HCP-established environments, and close connections to the medical and health sector. Access to health science information among HLIs followed an integrated online - offline pattern, with a notable gap between channel convenience and perceived authority. Importantly, self-rated health status showed a partial statistical mediation pattern in the association between HCP establishment and satisfaction with access channels in this cross-sectional study. These findings may help inform strategies to optimize access to health science information and improve HLIs training systems. Future efforts may prioritize official WeChat accounts as channels that are both authoritative and appealing for health communication. At the same time, strengthening HCP-related resource support and improving HLIs training in health information access and use may help translate knowledge into effective community health promotion practice.

## Data Availability

The original contributions presented in the study are included in the article/Supplementary material, further inquiries can be directed to the corresponding author.
