# Supplementary material for: Factors associated with access channels and satisfaction with health science popularization among health lifestyle instructors
Source: Front Public Health. 2026 Jul 29;14:1874724. doi: 10.3389/fpubh.2026.1874724 (PMC13463143; doi:10.3389/fpubh.2026.1874724)
Supplement: Supplementary file 1 [file Supplementary_file_1.pdf]

Questionnaire for Health Lifestyle Instructors in Nanjing

This questionnaire is designed to investigate your access channels to health science popularization and related satisfaction. All responses are anonymous and will only be used for academic research. Please answer truthfully based on your actual situation. The survey takes about 3-5 minutes to complete. Thank you for your cooperation!

Part 1: Socio-Demographic Information

1. Gender:

(1) Male

(2) Female

2. Date of Birth: ______ Year ______ Month ______ Day

3. Education Level:

(1) Junior high school or below

(2) Senior high school/secondary technical school

(3) College/Bachelor's degree

(4) Master's degree or above

4. Work unit：

(1)Municipal/District patriotic health campaign office/CDC/Health

promotion center

(2) Secondary/Tertiary hospital

(3) Community Health Service Center

(4) School/Enterprise

(5) Township/Community/Village

5. Smoking status:

(1) Smoker

(2) Non-smoker

6. Weight (kg): ______

7. Height (cm): ______

8. Self-Rated Overall Health Status:

(1) Very poor

(2) Poor

(3) General

(4) Good

(5) Very good

9. Whether Your Workplace Has Established a "Healthy Cell

Project" (e.g., Healthy District, Health-Promoting Hospital, Healthy Institution, etc.):

(1) Yes

(2) No

(3) Unknown

10. Connections with the Medical and Health Industry:

(1) I am engaged in this industry

(2) Family members are engaged in this industry

(3) Friends are engaged in this industry

(4) None

Part 2: Access Channels to Health Science Popularization

1.Which channels do you usually use to obtain health science popularization? 【Multiple Selection】

(1) Online searches

(2) Reading books/newspapers

(3) Community publicity (lectures, publicity corridors, leaflets)

(4) Consulting professional doctors

(5) Watching TV programs

(6) New media platforms

(7) Others (specify if other: ______)

2. Which of the above channels do you consider the most authoritative? 【Multiple Selection】

(1) Online searches

(2) Reading books/newspapers

(3) Community publicity (lectures, publicity corridors, leaflets)

(4) Consulting professional doctors

(5) Watching TV programs

(6) New media platforms

(7) Others (specify if other: ______)

3. Which of the above channels do you consider the most convenient? 【Multiple Selection】

(1) Online searches

(2) Reading books/newspapers

(3) Community publicity (lectures, publicity corridors, leaflets)

(4) Consulting professional doctors

(5) Watching TV programs

(6) New media platforms

(7) Others (specify if other: ______)

4. Which of the following new media platforms do you use to obtain health science popularization? 【Multiple Selection】

(1) WeChat official accounts

(2) Weibo

(3) News/Information apps (e.g., Headline, New Beijing News, etc.)

(4) Short-video apps (e.g., TikTok, Kuaishou, etc.)

(5) Regular video apps (e.g., Youku Video, Bilibili Video, etc.)

(6) Knowledge-based online communities (e.g., Zhihu, Douban, etc.)

(7) Online live streaming

(8) Others: _____

5. Which of the following new media platforms do you consider the most authoritative? 【Multiple Selection】

(1) WeChat official accounts

(2) Weibo

(3) News/Information apps (e.g., Headline, New Beijing News, etc.)

(4) Short-video apps (e.g., TikTok, Kuaishou, etc.)

(5) Regular video apps (e.g., Youku Video, Bilibili Video, etc.)

(6) Knowledge-based online communities (e.g., Zhihu, Douban, etc.)

(7) Online live streaming

(8) Others: _____

6. Which of the following new media platforms do you consider the most convenient? 【Multiple Selection】

(1) WeChat official accounts

(2) Weibo

(3) News/Information apps (e.g., Headline, New Beijing News, etc.)

(4) Short-video apps (e.g., TikTok, Kuaishou, etc.)

(5) Regular video apps (e.g., Youku Video, Bilibili Video, etc.)

(6) Knowledge-based online communities (e.g., Zhihu, Douban, etc.)

(7) Online live streaming

(8) Others: _____

7. Do you think the current channels for obtaining health science popularization can meet your needs? 【Single Selection】

(1) Yes

(2) No
